# Editors' Note

**DOI:** 10.5195/ijt.2021.6440

**Published:** 2021-12-16

**Authors:** Ellen R. Cohn, Jana Cason

## IJT'S INTERNATIONAL AUTHORSHIP AND READERSHIP

The *International Journal of Telerehabilitation* (IJT) is a biannual journal dedicated to advancing telerehabilitation by disseminating information about current research and practices. This open-source journal is indexed by PubMed and Scopus. IJT requires no readership or author fees.

IJT enjoys a diverse *international authorship.* In the current issue, we are very pleased to publish work by authors from Australia, Chile, India, Italy, Hong Kong, and Saudi Arabia, in addition to articles from the United States.

IJT also enjoys a wide *international readership.* Since its inception in 2008, approximately half of the journal's readership is from the United States. The journal's audience (in order of frequency) also includes readers from: Australia, India, Philippines, United Kingdom, Canada, Brazil, Italy, China, Spain, Germany, Singapore, France, South Korea, Turkey, Hong Kong, Ireland, Iran, Saudi Arabia, Chile, Netherlands, Malaysia, Finland, Indonesia, Columbia, Portugal, South Africa, Japan, Greece, Israel, Taiwan, Belgium, Thailand, Pakistan, Denmark, New Zealand, Austria, Poland, Sweden, Nigeria, Mexico, United Arab Emirates, Argentina, Switzerland, Peru, Norway, Russia, Puerto Rico, Oman, Egypt, Croatia, Czechia, Latvia, Romania, Kenya, Lithuania, Slovenia, Lebanon, Cyprus, Ukraine, Kuwait, Bangladesh, Jordan, Qatar, Estonia, Ghana, Bulgaria, Sri Lanka, Hungary, Iceland, Ecuador, Vietnam, Serbia, Bahrain, Malta, Uganda, Iraq, Bosnia and Herzegovina, Nepal, Tanzania, Guam, Palestine, Algeria, Morocco, Trinidad and Tobago, Georgia, Venezuela, Costa Rico, Ethiopia, Jamaica, Brunei, Cambodia, Bolivia, Maldives, Zimbabwe, Macao, North Macedonia, and Panama.

IJT does not actively engage in marketing. We surmise that this wide readership is testimony to the expanding global relevance of telemedicine, telehealth, and telerehabilitation.

Such a diverse international audience would not be likely without the journal's use of an open journal system (OJS) and the generous sponsorship of the University of Pittsburgh's Office of Scholarly Communication and Publishing University Library System.

## ISSUE OVERVIEW

As context, this issue was produced during an historic time. COVID-19, a global pandemic, is still dramatically affecting healthcare worldwide, as well as the global economy. Submissions to IJT have continued to increase, ostensibly to document how telerehabilitation is meeting those challenges. We regret that we are unable to publish the high volume of articles that are being submitted. Instead, we strive to promptly suggest other placements for work that is not central to telerehabilitation or relevant to the larger IJT audience.

The current issue of the multi-disciplinary *International Journal of Telerehabilitation* (IJT) features impactful research across rehabilitation disciplines, with many more articles about physical therapy (i.e., physiotherapy) than has been typical. The issue also includes the conference proceedings of an inter-professional educational effort that underscores that telerehabilitation at its best, is enriched by multi-disciplinary collaboration.

## CALL FOR SUBMISSIONS

We cordially invite submissions to the June 2022 issue beginning on January 1, 2022. IJT accepts original research, case studies, viewpoints, technology reviews, book reviews, and country reports that detail the status of telerehabilitation.

Sincerely,

Ellen R. Cohn, PhD, CCC-SLP, ASHA-F, IJT Editor

Jana Cason, DHSc, OTR/L, FAOTA, Senior Associate Editor

